# Bis{μ-[4-(1,3-benzothia­zol-2-yl)phen­yl]methane­thiol­ato-κ^4^ *S*,*S*′:*S*,*S*′}bis­[tricarbonyl­iron(I)](*Fe*—*Fe*)

**DOI:** 10.1107/S1600536812007581

**Published:** 2012-02-24

**Authors:** Shang Gao, Da-yong Jiang, Qing-cheng Liang, Qian Duan

**Affiliations:** a School of Materials Science and Engineering, Changchun University of Science and Technology, No. 7989 Weixing Road, Changchun 130022, People’s Republic of China

## Abstract

The title compound, [Fe_2_(C_14_H_10_NS_2_)_2_(CO)_6_], was synthesized as a structural and biochemical model for the active site of [FeFe]-hydrogenase. The bond lengths (Fe—Fe, Fe—S and Fe—C) and angles (C—Fe—Fe and Fe—S—Fe) are within expected ranges. The S⋯S distance [2.9069 (12) Å] and the dihedral angle between two Fe—S—Fe planes [78.5 (3)°] of the butterfly-shaped Fe_2_S_2_ core are enlarged compared with related bridged dithiol­ate diiron analogues. The calculated 4-benzothia­zolebenzyl best planes are almost parallel [dihedral angle = 3.7 (7)°].

## Related literature

For general background to [FeFe] hydrogenases, see: Cammack (1999 ▶); Evans & Pickett (2003 ▶); Peters *et al.* (1998 ▶); Nicolet *et al.* (1999 ▶); Si *et al.* (2008 ▶). For related structures and comparative geometric data, see: Tard & Pickett (2009 ▶). For the ligand synthesis, see: Palmer *et al.* (1971 ▶); Yoshino *et al.* (1986 ▶).
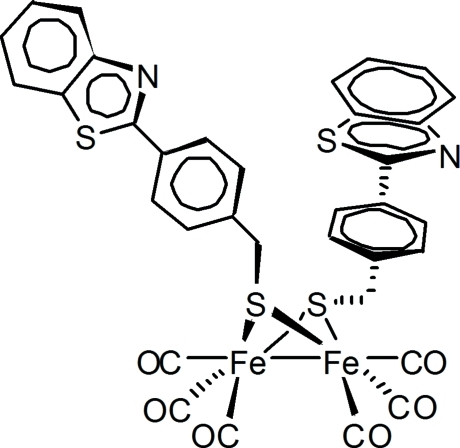


## Experimental

### 

#### Crystal data

[Fe_2_(C_14_H_10_NS_2_)_2_(CO)_6_]
*M* *_r_* = 792.46
Orthorhombic, 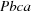

*a* = 12.8288 (14) Å
*b* = 16.8812 (17) Å
*c* = 31.089 (3) Å
*V* = 6732.8 (12) Å^3^
*Z* = 8
Mo *K*α radiation
μ = 1.16 mm^−1^
*T* = 273 K
0.33 × 0.29 × 0.11 mm

#### Data collection

Bruker SMART CCD diffractometer
Absorption correction: multi-scan (*SADABS*; Bruker, 1997 ▶) *T*_min_ = 0.703, *T*_max_ = 0.887
36569 measured reflections
6606 independent reflections
4253 reflections with *I* > 2σ(*I*)
*R*_int_ = 0.063

#### Refinement

*R*[*F* ^2^ > 2σ(*F* ^2^)] = 0.043
*wR*(*F* ^2^) = 0.107
*S* = 1.01
6606 reflections
433 parameters
H-atom parameters constrained
Δρ_max_ = 0.36 e Å^−3^
Δρ_min_ = −0.30 e Å^−3^

### 

Data collection: *SMART* (Bruker, 1997 ▶); cell refinement: *SAINT-Plus* (Bruker, 2001 ▶); data reduction: *SAINT-Plus*; program(s) used to solve structure: *SHELXS97* (Sheldrick, 2008 ▶); program(s) used to refine structure: *SHELXL97* (Sheldrick, 2008 ▶); molecular graphics: *SHELXTL* (Sheldrick, 2008 ▶); software used to prepare material for publication: *SHELXTL*.

## Supplementary Material

Crystal structure: contains datablock(s) global, I. DOI: 10.1107/S1600536812007581/vm2158sup1.cif

Structure factors: contains datablock(s) I. DOI: 10.1107/S1600536812007581/vm2158Isup2.hkl

Additional supplementary materials:  crystallographic information; 3D view; checkCIF report

## Figures and Tables

**Table:** 

Fe2—S2	2.2530 (9)
Fe2—S1	2.2704 (10)
Fe2—Fe1	2.5198 (7)
Fe1—S2	2.2529 (10)
Fe1—S1	2.2638 (10)

**Table:** 

C6—Fe2—Fe1	150.35 (14)
C1—Fe1—Fe2	150.49 (11)
Fe1—S2—Fe2	68.00 (3)
Fe1—S1—Fe2	67.52 (3)

**Fig. 1.**
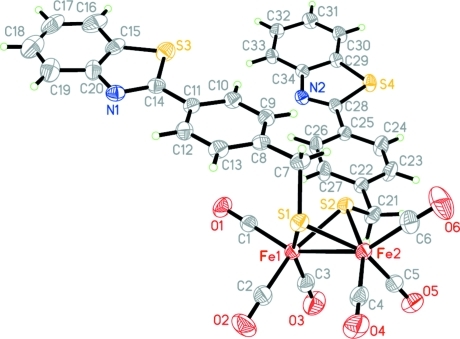
The molecular structure of the title compound, with displacement ellipsoids drawn at 30% probability level.

## supplementary crystallographic information

### Comment

The [FeFe] hydrogenases ([FeFe]Hases) are enzymes which can catalyze the reversible interconversion of protons to molecular hydrogen in nature (Cammack, 1999; Evans & Pickett, 2003). X-ray crystallography elucidated the active site of [FeFe]Hases (so-called H-cluster) as a 2Fe2S butterfly moiety in which a three-atom linker (—CH_2_XCH_2_—, *X* = CH_2_, NH or NH_2_^+^) bridged between the two S atoms (Peters *et al.*, 1998; Nicolet *et al.*, 1999). However, current research suggests that diiron complexes with non-bridged thiolate can also act as model for the H-cluster of [FeFe]Hases (Si *et al.*, 2008). We have synthesized the title compound as a structural model for the diiron subunit of the H-cluster. Herein we report its crystal structure. The title compound has a 2Fe2S core of butterfly conformation, and the Fe—Fe distance [2.5198 (7) Å] is within the expected range (Tard & Pickett, 2009). The two 4-benzothiazolebenzyl moieties reside in the conformation with the least steric hindrance in the molecule. As a result, the C1—Fe1—Fe2 [150.49 (11)°] angle and the C6—Fe2—Fe1 [150.35 (14)°] angle are almost equal. It is noteworthy that the length of S1···S2 [2.9069 (12) Å] and the dihedral angle between the planes defined by Fe1—S1—Fe2 and Fe1—S2—Fe2 [78.5 (3)°] are somewhat enlarged as compared with previously reported models with bridged dithiolate ligands (Tard & Pickett, 2009). The atoms of the 4-benzothiazolebenzyl moieties are almost coplanar with r.m.s. deviations of 0.0671 Å and 0.1115 Å respectively, and the dihedral angle between the two planes is 3.7 (7)°. Selected bond distances and angles are summarized in Table 1, and an *ORTEP* representation of the title compound is shown in Fig. 1.

### Experimental

The starting material 2-(4-bromomethylphenyl)-benzothiazole was prepared in 43% yield from 4-methylbenzonic acid and 2-aminophenthiol according to the literature procedure (Palmer *et al.*, 1971; Yoshino *et al.*, 1986). Super hydride LiEt_3_BH (1 *M* solution in THF, 8 ml, 8 mmol) was dropped into a degassed solution of (µ-S_2_)Fe_2_(CO)_6_ (1.38 g, 4 mmol) in dry THF (30 ml) by syringe at 195 K over 30 min. The mixture changed to dark emerald green. 2-(4-bromomethylphenyl)-benzothiazole (2.42 g, 4 mmol) was added to above solution, causing an immediate change in color to red. The reaction mixture was stirred for 2 h at 195 K, and an additional 1 h at room temperature. The solvent was removed on a rotary evaporator. The crude product was purified by column chromatography with silica by using CH_2_Cl_2_/hexane (1:10) as the eluent to give the title compound as a red solid (2.62 g, 85%). A single crystal suitable for X-ray study was obtained by slow evaporation of CH_2_Cl_2_/hexane (5:1, *v/v*) solution at room temperature.

### Refinement

Positional parameters of all the H atoms were calculated geometrically and were allowed to ride on the C atoms to which they are bonded, riding with C—H = 0.93 Å (aromatic) and 0.97 Å (methylene), with *U*_iso_(H) = 1.2*U*_eq_(C).

### Crystal data

**Table:**

[Fe_2_(C_14_H_10_NS_2_)_2_(CO)_6_]	*F*(000) = 3216
*M**_r_* = 792.46	*D*_x_ = 1.564 Mg m^−^^3^
Orthorhombic, *P**b**c**a*	Mo *K*α radiation, λ = 0.71073 Å
Hall symbol: -P 2ac 2ab	Cell parameters from 4686 reflections
*a* = 12.8288 (14) Å	θ = 2.4–23.0°
*b* = 16.8812 (17) Å	µ = 1.16 mm^−^^1^
*c* = 31.089 (3) Å	*T* = 273 K
*V* = 6732.8 (12) Å^3^	Block, red
*Z* = 8	0.33 × 0.29 × 0.11 mm

### Data collection

**Table:**

Bruker SMART CCD diffractometer	6606 independent reflections
Radiation source: fine-focus sealed tube	4253 reflections with *I* > 2σ(*I*)
Graphite monochromator	*R*_int_ = 0.063
φ and ω scans	θ_max_ = 26.0°, θ_min_ = 2.1°
Absorption correction: multi-scan (*SADABS*; Bruker, 1997)	*h* = −15→15
*T*_min_ = 0.703, *T*_max_ = 0.887	*k* = −20→20
36569 measured reflections	*l* = −38→38

### Refinement

**Table:**

Refinement on *F*^2^	0 restraints
Least-squares matrix: full	H-atom parameters constrained
*R*[*F*^2^ > 2σ(*F*^2^)] = 0.043	*w* = 1/[σ^2^(*F*_o_^2^) + (0.0517*P*)^2^] where *P* = (*F*_o_^2^ + 2*F*_c_^2^)/3
*wR*(*F*^2^) = 0.107	(Δ/σ)_max_ < 0.001
*S* = 1.01	Δρ_max_ = 0.36 e Å^−^^3^
6606 reflections	Δρ_min_ = −0.30 e Å^−^^3^
433 parameters	

### Special details

**Table:**

Geometry. All e.s.d.'s (except the e.s.d. in the dihedral angle between two l.s. planes) are estimated using the full covariance matrix. The cell e.s.d.'s are taken into account individually in the estimation of e.s.d.'s in distances, angles and torsion angles; correlations between e.s.d.'s in cell parameters are only used when they are defined by crystal symmetry. An approximate (isotropic) treatment of cell e.s.d.'s is used for estimating e.s.d.'s involving l.s. planes.
Refinement. Refinement of *F*^2^ against ALL reflections. The weighted *R*-factor *wR* and goodness of fit *S* are based on *F*^2^, conventional *R*-factors *R* are based on *F*, with *F* set to zero for negative *F*^2^. The threshold expression of *F*^2^ > σ(*F*^2^) is used only for calculating *R*-factors(gt) *etc*. and is not relevant to the choice of reflections for refinement. *R*-factors based on *F*^2^ are statistically about twice as large as those based on *F*, and *R*-factors based on ALL data will be even larger.

### Fractional atomic coordinates and isotropic or equivalent isotropic displacement parameters (Å^2^)

**Table:**

	*x*	*y*	*z*	*U*_iso_*/*U*_eq_	
Fe2	0.41571 (4)	0.11737 (3)	0.195948 (15)	0.04572 (15)	
Fe1	0.45475 (4)	0.25060 (3)	0.163154 (14)	0.04442 (14)	
S2	0.29985 (6)	0.18857 (5)	0.15759 (3)	0.0448 (2)	
S4	−0.24312 (6)	0.36861 (5)	0.08716 (3)	0.0524 (2)	
S1	0.50722 (7)	0.13427 (5)	0.13412 (3)	0.0504 (2)	
S3	0.31365 (8)	0.35719 (6)	−0.07790 (3)	0.0672 (3)	
N2	−0.08397 (19)	0.44143 (15)	0.05458 (9)	0.0436 (6)	
N1	0.5187 (2)	0.35694 (16)	−0.07125 (9)	0.0529 (7)	
O1	0.4457 (2)	0.36163 (16)	0.09040 (8)	0.0749 (8)	
O5	0.3230 (2)	0.16903 (17)	0.27661 (8)	0.0726 (8)	
C34	−0.1709 (2)	0.46479 (18)	0.03111 (10)	0.0418 (7)	
C26	0.0706 (3)	0.3589 (2)	0.10309 (12)	0.0586 (10)	
H26A	0.0918	0.3901	0.0800	0.070*	
C10	0.3359 (3)	0.2510 (2)	0.00364 (10)	0.0511 (9)	
H10A	0.2727	0.2715	−0.0058	0.061*	
C11	0.4281 (3)	0.2747 (2)	−0.01612 (10)	0.0455 (8)	
C7	0.4314 (3)	0.1043 (2)	0.08637 (10)	0.0542 (9)	
H7A	0.4605	0.0556	0.0749	0.065*	
H7B	0.3602	0.0934	0.0950	0.065*	
C25	−0.0334 (2)	0.35275 (18)	0.11261 (10)	0.0423 (8)	
C22	0.1166 (2)	0.27463 (19)	0.16205 (10)	0.0440 (8)	
C18	0.5292 (6)	0.4863 (3)	−0.16317 (16)	0.1007 (18)	
H18A	0.5783	0.5118	−0.1803	0.121*	
C32	−0.2597 (3)	0.5322 (2)	−0.02509 (11)	0.0530 (9)	
H32A	−0.2593	0.5663	−0.0486	0.064*	
C30	−0.3571 (3)	0.4472 (2)	0.02233 (12)	0.0560 (9)	
H30A	−0.4197	0.4244	0.0309	0.067*	
C29	−0.2650 (2)	0.43106 (18)	0.04393 (10)	0.0437 (8)	
O3	0.3866 (3)	0.35598 (18)	0.23209 (10)	0.0979 (11)	
C28	−0.1102 (2)	0.39109 (18)	0.08425 (10)	0.0422 (8)	
C13	0.5225 (3)	0.1903 (2)	0.03241 (11)	0.0551 (9)	
H13A	0.5857	0.1702	0.0422	0.066*	
C24	−0.0616 (3)	0.3060 (2)	0.14676 (12)	0.0668 (11)	
H24A	−0.1318	0.3001	0.1535	0.080*	
C14	0.4290 (3)	0.3283 (2)	−0.05351 (10)	0.0495 (9)	
C33	−0.1685 (3)	0.51657 (19)	−0.00389 (10)	0.0481 (8)	
H33A	−0.1063	0.5399	−0.0126	0.058*	
C2	0.5871 (3)	0.2690 (2)	0.17839 (13)	0.0652 (11)	
C4	0.5353 (3)	0.0884 (2)	0.22054 (12)	0.0605 (10)	
C21	0.1941 (3)	0.2310 (2)	0.18951 (11)	0.0552 (9)	
H21A	0.1587	0.1889	0.2049	0.066*	
H21B	0.2231	0.2671	0.2106	0.066*	
C3	0.4120 (3)	0.3152 (2)	0.20467 (13)	0.0625 (10)	
C1	0.4491 (3)	0.3187 (2)	0.11812 (12)	0.0526 (9)	
O4	0.6126 (2)	0.06903 (19)	0.23580 (10)	0.0921 (10)	
C31	−0.3530 (3)	0.4977 (2)	−0.01198 (12)	0.0586 (10)	
H31A	−0.4139	0.5093	−0.0269	0.070*	
C9	0.3376 (3)	0.19729 (19)	0.03703 (11)	0.0513 (8)	
H9A	0.2754	0.1821	0.0500	0.062*	
C8	0.4305 (3)	0.16578 (18)	0.05156 (10)	0.0442 (8)	
C15	0.3840 (4)	0.4116 (2)	−0.11322 (11)	0.0607 (10)	
C12	0.5216 (3)	0.2437 (2)	−0.00070 (11)	0.0552 (9)	
H12A	0.5842	0.2595	−0.0131	0.066*	
C5	0.3592 (3)	0.1476 (2)	0.24554 (12)	0.0521 (9)	
C19	0.5647 (4)	0.4416 (2)	−0.13029 (15)	0.0860 (14)	
H19A	0.6357	0.4365	−0.1248	0.103*	
C20	0.4908 (4)	0.4033 (2)	−0.10481 (13)	0.0651 (11)	
C17	0.4271 (6)	0.4967 (3)	−0.17299 (14)	0.0899 (16)	
H17A	0.4084	0.5284	−0.1962	0.108*	
C6	0.3551 (3)	0.0222 (2)	0.19312 (14)	0.0721 (11)	
C23	0.0119 (3)	0.2678 (2)	0.17106 (11)	0.0653 (11)	
H23A	−0.0093	0.2367	0.1941	0.078*	
O2	0.6696 (2)	0.2827 (2)	0.18933 (12)	0.1070 (12)	
O6	0.3127 (3)	−0.0373 (2)	0.19280 (14)	0.1274 (14)	
C27	0.1442 (3)	0.3195 (2)	0.12730 (12)	0.0627 (10)	
H27A	0.2142	0.3236	0.1198	0.075*	
C16	0.3491 (5)	0.4594 (3)	−0.14816 (14)	0.0909 (15)	
H16A	0.2787	0.4658	−0.1543	0.109*	

### Atomic displacement parameters (Å^2^)

**Table:**

	*U*^11^	*U*^22^	*U*^33^	*U*^12^	*U*^13^	*U*^23^
Fe2	0.0463 (3)	0.0436 (3)	0.0473 (3)	0.0032 (2)	−0.0052 (2)	0.0097 (2)
Fe1	0.0438 (3)	0.0442 (3)	0.0453 (3)	−0.0008 (2)	−0.0045 (2)	0.0079 (2)
S2	0.0391 (4)	0.0502 (5)	0.0450 (5)	0.0035 (4)	−0.0039 (4)	0.0069 (4)
S4	0.0335 (5)	0.0582 (5)	0.0654 (6)	0.0032 (4)	0.0053 (4)	0.0145 (4)
S1	0.0468 (5)	0.0548 (5)	0.0497 (5)	0.0115 (4)	−0.0017 (4)	0.0066 (4)
S3	0.0691 (7)	0.0710 (6)	0.0615 (6)	0.0076 (6)	−0.0029 (5)	0.0073 (5)
N2	0.0304 (14)	0.0461 (15)	0.0545 (17)	0.0023 (12)	−0.0031 (13)	0.0029 (14)
N1	0.062 (2)	0.0453 (16)	0.0514 (17)	0.0024 (15)	−0.0078 (15)	−0.0050 (14)
O1	0.104 (2)	0.0622 (17)	0.0582 (16)	−0.0036 (15)	−0.0052 (16)	0.0207 (14)
O5	0.0732 (19)	0.090 (2)	0.0545 (16)	0.0136 (16)	0.0002 (14)	0.0010 (15)
C34	0.0357 (18)	0.0420 (18)	0.0478 (19)	0.0040 (15)	0.0022 (15)	−0.0049 (15)
C26	0.039 (2)	0.072 (2)	0.066 (2)	−0.0012 (18)	−0.0001 (18)	0.029 (2)
C10	0.0430 (19)	0.059 (2)	0.051 (2)	0.0071 (17)	−0.0040 (17)	0.0003 (18)
C11	0.044 (2)	0.0516 (19)	0.0405 (18)	0.0024 (16)	−0.0014 (16)	−0.0032 (16)
C7	0.062 (2)	0.052 (2)	0.049 (2)	0.0084 (18)	−0.0028 (18)	−0.0063 (17)
C25	0.0339 (18)	0.0472 (19)	0.0458 (19)	0.0031 (15)	0.0021 (15)	0.0017 (16)
C22	0.0383 (18)	0.0501 (19)	0.0434 (19)	0.0043 (15)	0.0026 (15)	0.0030 (16)
C18	0.171 (6)	0.057 (3)	0.074 (3)	−0.003 (4)	0.018 (4)	−0.003 (3)
C32	0.046 (2)	0.058 (2)	0.056 (2)	0.0072 (18)	−0.0039 (18)	0.0101 (17)
C30	0.0302 (18)	0.060 (2)	0.077 (3)	0.0014 (17)	−0.0019 (18)	0.006 (2)
C29	0.0338 (18)	0.0416 (18)	0.056 (2)	0.0059 (15)	0.0023 (16)	0.0022 (15)
O3	0.146 (3)	0.0724 (19)	0.075 (2)	0.000 (2)	0.017 (2)	−0.0111 (17)
C28	0.0319 (17)	0.0424 (18)	0.052 (2)	0.0024 (15)	0.0015 (15)	0.0002 (16)
C13	0.043 (2)	0.067 (2)	0.055 (2)	0.0128 (18)	−0.0048 (17)	−0.0011 (19)
C24	0.0332 (19)	0.098 (3)	0.070 (3)	0.010 (2)	0.0112 (18)	0.026 (2)
C14	0.053 (2)	0.049 (2)	0.047 (2)	0.0033 (17)	−0.0008 (17)	−0.0102 (16)
C33	0.0404 (19)	0.048 (2)	0.056 (2)	−0.0008 (16)	0.0039 (17)	0.0056 (17)
C2	0.059 (3)	0.065 (2)	0.072 (3)	−0.004 (2)	−0.011 (2)	0.021 (2)
C4	0.059 (3)	0.065 (2)	0.058 (2)	0.005 (2)	−0.001 (2)	0.0179 (19)
C21	0.048 (2)	0.071 (2)	0.047 (2)	0.0098 (18)	0.0031 (17)	0.0138 (18)
C3	0.078 (3)	0.052 (2)	0.057 (2)	−0.005 (2)	−0.001 (2)	0.009 (2)
C1	0.056 (2)	0.050 (2)	0.052 (2)	−0.0021 (18)	−0.0042 (18)	0.0022 (18)
O4	0.066 (2)	0.122 (3)	0.089 (2)	0.0244 (19)	−0.0154 (17)	0.0322 (19)
C31	0.041 (2)	0.061 (2)	0.073 (3)	0.0081 (18)	−0.0098 (19)	0.008 (2)
C9	0.043 (2)	0.059 (2)	0.051 (2)	−0.0027 (17)	−0.0011 (17)	0.0003 (18)
C8	0.048 (2)	0.0437 (18)	0.0409 (18)	0.0069 (16)	−0.0026 (16)	−0.0073 (15)
C15	0.097 (3)	0.045 (2)	0.040 (2)	0.009 (2)	0.000 (2)	−0.0059 (17)
C12	0.047 (2)	0.066 (2)	0.053 (2)	−0.0006 (19)	0.0058 (17)	−0.0018 (19)
C5	0.051 (2)	0.051 (2)	0.055 (2)	0.0039 (18)	−0.0095 (19)	0.0114 (18)
C19	0.108 (4)	0.061 (3)	0.089 (3)	−0.003 (3)	0.026 (3)	−0.003 (3)
C20	0.082 (3)	0.047 (2)	0.066 (3)	−0.013 (2)	0.028 (2)	−0.015 (2)
C17	0.169 (6)	0.054 (3)	0.047 (3)	0.007 (3)	0.004 (3)	0.005 (2)
C6	0.076 (3)	0.058 (3)	0.082 (3)	−0.003 (2)	0.004 (2)	0.008 (2)
C23	0.046 (2)	0.096 (3)	0.054 (2)	0.009 (2)	0.0088 (18)	0.030 (2)
O2	0.0578 (19)	0.124 (3)	0.139 (3)	−0.0199 (19)	−0.039 (2)	0.031 (2)
O6	0.137 (3)	0.066 (2)	0.179 (4)	−0.032 (2)	0.025 (3)	−0.014 (2)
C27	0.0312 (19)	0.082 (3)	0.075 (3)	0.0012 (19)	0.0035 (18)	0.030 (2)
C16	0.143 (5)	0.068 (3)	0.062 (3)	0.022 (3)	−0.011 (3)	−0.013 (2)

### Geometric parameters (Å, º)

**Table:**

Fe2—C5	1.779 (4)	C22—C27	1.366 (4)
Fe2—C4	1.783 (4)	C22—C23	1.376 (5)
Fe2—C6	1.786 (4)	C22—C21	1.504 (4)
Fe2—S2	2.2530 (9)	C18—C19	1.350 (6)
Fe2—S1	2.2704 (10)	C18—C17	1.356 (7)
Fe2—Fe1	2.5198 (7)	C18—H18A	0.9300
Fe1—C3	1.776 (4)	C32—C33	1.368 (4)
Fe1—C2	1.790 (4)	C32—C31	1.392 (5)
Fe1—C1	1.812 (4)	C32—H32A	0.9300
Fe1—S2	2.2529 (10)	C30—C31	1.367 (5)
Fe1—S1	2.2638 (10)	C30—C29	1.386 (4)
S2—C21	1.826 (3)	C30—H30A	0.9300
S4—C29	1.731 (3)	O3—C3	1.143 (4)
S4—C28	1.749 (3)	C13—C12	1.368 (5)
S1—C7	1.845 (3)	C13—C8	1.385 (4)
S3—C15	1.692 (4)	C13—H13A	0.9300
S3—C14	1.733 (4)	C24—C23	1.369 (5)
N2—C28	1.299 (4)	C24—H24A	0.9300
N2—C34	1.390 (4)	C33—H33A	0.9300
N1—C20	1.353 (5)	C2—O2	1.136 (4)
N1—C14	1.364 (4)	C4—O4	1.146 (4)
O1—C1	1.127 (4)	C21—H21A	0.9700
O5—C5	1.131 (4)	C21—H21B	0.9700
C34—C29	1.393 (4)	C31—H31A	0.9300
C34—C33	1.396 (4)	C9—C8	1.382 (4)
C26—C25	1.371 (4)	C9—H9A	0.9300
C26—C27	1.378 (4)	C15—C20	1.403 (6)
C26—H26A	0.9300	C15—C16	1.425 (5)
C10—C9	1.378 (4)	C12—H12A	0.9300
C10—C11	1.391 (4)	C19—C20	1.394 (5)
C10—H10A	0.9300	C19—H19A	0.9300
C11—C12	1.394 (5)	C17—C16	1.411 (7)
C11—C14	1.473 (5)	C17—H17A	0.9300
C7—C8	1.500 (4)	C6—O6	1.143 (4)
C7—H7A	0.9700	C23—H23A	0.9300
C7—H7B	0.9700	C27—H27A	0.9300
C25—C24	1.372 (4)	C16—H16A	0.9300
C25—C28	1.472 (4)		
			
C5—Fe2—C4	93.33 (16)	C33—C32—H32A	119.5
C5—Fe2—C6	97.07 (18)	C31—C32—H32A	119.5
C4—Fe2—C6	98.57 (18)	C31—C30—C29	117.9 (3)
C5—Fe2—S2	92.10 (11)	C31—C30—H30A	121.1
C4—Fe2—S2	160.24 (12)	C29—C30—H30A	121.1
C6—Fe2—S2	99.59 (14)	C30—C29—C34	121.3 (3)
C5—Fe2—S1	155.33 (11)	C30—C29—S4	129.4 (3)
C4—Fe2—S1	87.27 (12)	C34—C29—S4	109.3 (2)
C6—Fe2—S1	107.23 (14)	N2—C28—C25	122.7 (3)
S2—Fe2—S1	79.98 (3)	N2—C28—S4	115.6 (2)
C5—Fe2—Fe1	100.12 (11)	C25—C28—S4	121.7 (2)
C4—Fe2—Fe1	104.31 (12)	C12—C13—C8	120.8 (3)
C6—Fe2—Fe1	150.35 (14)	C12—C13—H13A	119.6
S2—Fe2—Fe1	56.00 (3)	C8—C13—H13A	119.6
S1—Fe2—Fe1	56.11 (3)	C23—C24—C25	121.1 (3)
C3—Fe1—C2	89.66 (19)	C23—C24—H24A	119.5
C3—Fe1—C1	99.19 (16)	C25—C24—H24A	119.5
C2—Fe1—C1	97.62 (16)	N1—C14—C11	123.0 (3)
C3—Fe1—S2	93.94 (13)	N1—C14—S3	116.3 (3)
C2—Fe1—S2	159.67 (12)	C11—C14—S3	120.7 (3)
C1—Fe1—S2	101.52 (11)	C32—C33—C34	118.5 (3)
C3—Fe1—S1	156.07 (12)	C32—C33—H33A	120.7
C2—Fe1—S1	88.51 (14)	C34—C33—H33A	120.7
C1—Fe1—S1	104.71 (11)	O2—C2—Fe1	177.2 (4)
S2—Fe1—S1	80.12 (3)	O4—C4—Fe2	178.9 (4)
C3—Fe1—Fe2	101.10 (12)	C22—C21—S2	112.0 (2)
C2—Fe1—Fe2	103.68 (12)	C22—C21—H21A	109.2
C1—Fe1—Fe2	150.49 (11)	S2—C21—H21A	109.2
S2—Fe1—Fe2	56.00 (3)	C22—C21—H21B	109.2
S1—Fe1—Fe2	56.36 (3)	S2—C21—H21B	109.2
C21—S2—Fe1	115.54 (13)	H21A—C21—H21B	107.9
C21—S2—Fe2	114.28 (11)	O3—C3—Fe1	178.1 (4)
Fe1—S2—Fe2	68.00 (3)	O1—C1—Fe1	179.3 (3)
C29—S4—C28	89.18 (15)	C30—C31—C32	121.5 (3)
C7—S1—Fe1	113.71 (11)	C30—C31—H31A	119.2
C7—S1—Fe2	111.97 (12)	C32—C31—H31A	119.2
Fe1—S1—Fe2	67.52 (3)	C10—C9—C8	120.9 (3)
C15—S3—C14	88.95 (19)	C10—C9—H9A	119.6
C28—N2—C34	110.5 (3)	C8—C9—H9A	119.6
C20—N1—C14	107.1 (3)	C9—C8—C13	118.7 (3)
N2—C34—C29	115.4 (3)	C9—C8—C7	120.6 (3)
N2—C34—C33	124.7 (3)	C13—C8—C7	120.7 (3)
C29—C34—C33	119.9 (3)	C20—C15—C16	120.3 (4)
C25—C26—C27	120.8 (3)	C20—C15—S3	110.2 (3)
C25—C26—H26A	119.6	C16—C15—S3	129.4 (4)
C27—C26—H26A	119.6	C13—C12—C11	120.9 (3)
C9—C10—C11	120.6 (3)	C13—C12—H12A	119.5
C9—C10—H10A	119.7	C11—C12—H12A	119.5
C11—C10—H10A	119.7	O5—C5—Fe2	178.0 (3)
C10—C11—C12	118.1 (3)	C18—C19—C20	117.4 (5)
C10—C11—C14	122.2 (3)	C18—C19—H19A	121.3
C12—C11—C14	119.7 (3)	C20—C19—H19A	121.3
C8—C7—S1	113.2 (2)	N1—C20—C19	121.8 (4)
C8—C7—H7A	108.9	N1—C20—C15	117.4 (3)
S1—C7—H7A	108.9	C19—C20—C15	120.9 (4)
C8—C7—H7B	108.9	C18—C17—C16	120.3 (5)
S1—C7—H7B	108.9	C18—C17—H17A	119.9
H7A—C7—H7B	107.7	C16—C17—H17A	119.9
C26—C25—C24	117.9 (3)	O6—C6—Fe2	176.5 (4)
C26—C25—C28	119.3 (3)	C24—C23—C22	121.4 (3)
C24—C25—C28	122.7 (3)	C24—C23—H23A	119.3
C27—C22—C23	117.4 (3)	C22—C23—H23A	119.3
C27—C22—C21	123.3 (3)	C22—C27—C26	121.5 (3)
C23—C22—C21	119.3 (3)	C22—C27—H27A	119.2
C19—C18—C17	124.6 (6)	C26—C27—H27A	119.2
C19—C18—H18A	117.7	C17—C16—C15	116.6 (5)
C17—C18—H18A	117.7	C17—C16—H16A	121.7
C33—C32—C31	120.9 (3)	C15—C16—H16A	121.7
			
C5—Fe2—Fe1—C3	1.59 (18)	C31—C30—C29—C34	−0.3 (5)
C4—Fe2—Fe1—C3	−94.52 (19)	C31—C30—C29—S4	−178.2 (3)
C6—Fe2—Fe1—C3	126.1 (3)	N2—C34—C29—C30	−177.8 (3)
S2—Fe2—Fe1—C3	87.26 (14)	C33—C34—C29—C30	0.7 (5)
S1—Fe2—Fe1—C3	−171.20 (14)	N2—C34—C29—S4	0.5 (3)
C5—Fe2—Fe1—C2	93.97 (18)	C33—C34—C29—S4	178.9 (2)
C4—Fe2—Fe1—C2	−2.14 (19)	C28—S4—C29—C30	177.2 (3)
C6—Fe2—Fe1—C2	−141.5 (3)	C28—S4—C29—C34	−0.9 (2)
S2—Fe2—Fe1—C2	179.64 (15)	C34—N2—C28—C25	176.6 (3)
S1—Fe2—Fe1—C2	−78.81 (15)	C34—N2—C28—S4	−1.1 (3)
C5—Fe2—Fe1—C1	−131.0 (3)	C26—C25—C28—N2	−11.7 (5)
C4—Fe2—Fe1—C1	132.9 (3)	C24—C25—C28—N2	173.1 (3)
C6—Fe2—Fe1—C1	−6.5 (4)	C26—C25—C28—S4	165.9 (3)
S2—Fe2—Fe1—C1	−45.4 (2)	C24—C25—C28—S4	−9.3 (5)
S1—Fe2—Fe1—C1	56.2 (2)	C29—S4—C28—N2	1.2 (3)
C5—Fe2—Fe1—S2	−85.67 (12)	C29—S4—C28—C25	−176.5 (3)
C4—Fe2—Fe1—S2	178.22 (13)	C26—C25—C24—C23	1.2 (6)
C6—Fe2—Fe1—S2	38.8 (3)	C28—C25—C24—C23	176.5 (4)
S1—Fe2—Fe1—S2	101.55 (4)	C20—N1—C14—C11	179.6 (3)
C5—Fe2—Fe1—S1	172.78 (12)	C20—N1—C14—S3	0.5 (3)
C4—Fe2—Fe1—S1	76.68 (13)	C10—C11—C14—N1	174.9 (3)
C6—Fe2—Fe1—S1	−62.7 (3)	C12—C11—C14—N1	−7.7 (5)
S2—Fe2—Fe1—S1	−101.55 (4)	C10—C11—C14—S3	−6.1 (5)
C3—Fe1—S2—C21	6.63 (16)	C12—C11—C14—S3	171.3 (3)
C2—Fe1—S2—C21	106.4 (4)	C15—S3—C14—N1	−1.0 (3)
C1—Fe1—S2—C21	−93.59 (16)	C15—S3—C14—C11	179.9 (3)
S1—Fe1—S2—C21	163.26 (12)	C31—C32—C33—C34	0.4 (5)
Fe2—Fe1—S2—C21	107.37 (12)	N2—C34—C33—C32	177.6 (3)
C3—Fe1—S2—Fe2	−100.74 (12)	C29—C34—C33—C32	−0.7 (5)
C2—Fe1—S2—Fe2	−1.0 (4)	C27—C22—C21—S2	−38.0 (4)
C1—Fe1—S2—Fe2	159.04 (12)	C23—C22—C21—S2	139.9 (3)
S1—Fe1—S2—Fe2	55.89 (3)	Fe1—S2—C21—C22	106.5 (2)
C5—Fe2—S2—C21	−8.34 (18)	Fe2—S2—C21—C22	−177.4 (2)
C4—Fe2—S2—C21	−114.2 (4)	C29—C30—C31—C32	0.0 (5)
C6—Fe2—S2—C21	89.20 (19)	C33—C32—C31—C30	−0.1 (6)
S1—Fe2—S2—C21	−164.82 (14)	C11—C10—C9—C8	−0.1 (5)
Fe1—Fe2—S2—C21	−109.14 (14)	C10—C9—C8—C13	1.2 (5)
C5—Fe2—S2—Fe1	100.80 (11)	C10—C9—C8—C7	−176.8 (3)
C4—Fe2—S2—Fe1	−5.1 (4)	C12—C13—C8—C9	−1.0 (5)
C6—Fe2—S2—Fe1	−161.66 (14)	C12—C13—C8—C7	177.0 (3)
S1—Fe2—S2—Fe1	−55.69 (3)	S1—C7—C8—C9	−121.3 (3)
C3—Fe1—S1—C7	126.8 (4)	S1—C7—C8—C13	60.7 (4)
C2—Fe1—S1—C7	−147.36 (18)	C14—S3—C15—C20	1.1 (3)
C1—Fe1—S1—C7	−49.88 (18)	C14—S3—C15—C16	179.7 (4)
S2—Fe1—S1—C7	49.56 (13)	C8—C13—C12—C11	−0.2 (5)
Fe2—Fe1—S1—C7	105.09 (13)	C10—C11—C12—C13	1.2 (5)
C3—Fe1—S1—Fe2	21.7 (3)	C14—C11—C12—C13	−176.3 (3)
C2—Fe1—S1—Fe2	107.54 (12)	C17—C18—C19—C20	0.2 (7)
C1—Fe1—S1—Fe2	−154.97 (12)	C14—N1—C20—C19	−179.5 (3)
S2—Fe1—S1—Fe2	−55.54 (3)	C14—N1—C20—C15	0.4 (4)
C5—Fe2—S1—C7	−124.8 (3)	C18—C19—C20—N1	179.7 (4)
C4—Fe2—S1—C7	143.13 (17)	C18—C19—C20—C15	−0.2 (6)
C6—Fe2—S1—C7	44.99 (19)	C16—C15—C20—N1	−179.8 (3)
S2—Fe2—S1—C7	−52.03 (12)	S3—C15—C20—N1	−1.1 (4)
Fe1—Fe2—S1—C7	−107.60 (12)	C16—C15—C20—C19	0.1 (5)
C5—Fe2—S1—Fe1	−17.2 (3)	S3—C15—C20—C19	178.8 (3)
C4—Fe2—S1—Fe1	−109.28 (13)	C19—C18—C17—C16	−0.1 (8)
C6—Fe2—S1—Fe1	152.59 (15)	C25—C24—C23—C22	−0.3 (6)
S2—Fe2—S1—Fe1	55.57 (3)	C27—C22—C23—C24	−1.4 (6)
C28—N2—C34—C29	0.4 (4)	C21—C22—C23—C24	−179.5 (4)
C28—N2—C34—C33	−177.9 (3)	C23—C22—C27—C26	2.3 (6)
C9—C10—C11—C12	−1.1 (5)	C21—C22—C27—C26	−179.7 (3)
C9—C10—C11—C14	176.4 (3)	C25—C26—C27—C22	−1.5 (6)
Fe1—S1—C7—C8	55.7 (3)	C18—C17—C16—C15	−0.1 (6)
Fe2—S1—C7—C8	129.9 (2)	C20—C15—C16—C17	0.1 (5)
C27—C26—C25—C24	−0.3 (6)	S3—C15—C16—C17	−178.4 (3)
C27—C26—C25—C28	−175.7 (3)		
